# Role of inflammation-related genes as prognostic biomarkers and mechanistic implications in idiopathic pulmonary fibrosis

**DOI:** 10.3389/fgene.2025.1602588

**Published:** 2025-06-18

**Authors:** Bing Bai, Wenfei Zhao, Fazhan Li, Yang Mi, Pengyuan Zheng

**Affiliations:** ^1^ Fuhua Street Branch of the Fifth Affiliated Hospital of Zhengzhou University, Zhengzhou, Henan, China; ^2^ Henan Key Laboratory of Helicobacter pylori and Microbiota and Gastrointestinal Cancer, Marshall Medical Research Center, The Fifth Affiliated Hospital of Zhengzhou University, Zhengzhou, China; ^3^ Department of General Practice, Zhengzhou Fuhua Street Community Health Service Center, Zhengzhou, Henan, China; ^4^ Department of Respiratory and Critical Care Medicine, The Fifth Affiliated Hospital of Zhengzhou University, Zhengzhou, Henan, China

**Keywords:** idiopathic pulmonary fibrosis, CCL2, inflammation-related genes, prognostic model, immune cell infiltration, biomarker

## Abstract

**Introduction:**

Idiopathic Pulmonary Fibrosis (IPF) is a chronic, progressive lung disorder characterized by excessive fibrosis and structural remodeling of lung tissue. The role of inflammation in developing and progressing IPF is increasingly recognized as critical. However, the precise mechanisms and pathways of inflammation in IPF remain unclear. This study aimed to identify inflammation-related genes in IPF and develop a prognostic risk model using machine learning approaches.

**Methods:**

The IPF dataset GSE70866 from the Gene Expression Omnibus database was analyzed to identify inflammation-related genes. Unsupervised clustering algorithms were used to classify IPF samples, followed by weighted gene co-expression network analysis (WGCNA) to identify highly correlated genes. Least absolute shrinkage and selection operator (LASSO) regression was then applied, and the intersection of results pinpointed critical hub genes, primarily *CCL2* and *STAB1*. A rat model of pulmonary fibrosis was established, and lentivirus transfection was used to knock down *CCL2* expression. The transfection effect and hub gene expression were validated using Quantitative polymerase chain reaction, Western blot, immunohistochemistry, enzyme-linked immunosorbent assay, hematoxylin-eosin staining, and Masson’s trichrome staining. Levels of α-SMA and COL1A1 were also assessed.

**Results:**

WGCNA and LASSO regression analyses identified *CCL2* and *STAB1* as significant contributors to IPF, closely associated with patient prognosis and immune cell infiltration. Protein-protein interaction network analysis established *CCL2* as a novel biomarker for IPF. In a rat model of IPF, *CCL2* expression was significantly elevated compared to that in the controls. Knockdown of *CCL2* expression alleviated pulmonary fibrosis and reduced the expression of COL1A1 protein and α-SMA protein. *CCL2* promotes the expression of COL1A1 protein and α-SMA proteins, suggesting that the mechanism of inflammation-induced pulmonary fibrosis may involve the regulation of COL1A1 and α-SMA by *CCL2*.

**Discussion:**

These findings establish *CCL2* as a promising biomarker and potential therapeutic target for IPF.

## Introduction

Idiopathic pulmonary fibrosis (IPF) is a chronic, progressive fibrotic lung disease characterized by destruction of the alveoli and remodeling of the interstitial lung structures. Pathologically, IPF is defined by fibrosis and structural alterations of lung tissue. Clinically, it manifests as thickening of the alveolar walls, reduced alveolar volume, and decreased lung elasticity (Richeldi et al., 2017). Patients with IPF commonly present with symptoms including persistent dry cough, fatigue, and progressively worsening dyspnea on exertion. As the disease advances, severe complications such as respiratory failure may arise (Glass et al., 2022). Despite extensive research efforts, the precise etiology of IPF remains elusive. Current therapeutic strategies primarily target pathological processes, aiming to reduce inflammation and manage fibrosis (Munchel and Shea, 2021; Abuserewa et al., 2021). Given the irreversible nature of IPF and the difficulties in halting its progression, elucidating its pathogenesis and identifying novel biomarkers for diagnosis, therapy, and prognosis are of critical importance.

Accumulating evidence indicates that inflammation is involved at multiple stages of IPF, playing a pivotal role in disease initiation and progression (Mura et al., 2005). Lung tissues from IPF patients exhibit mild inflammatory responses, characterized by the presence of various pro-inflammatory mediators and immune cell infiltration (Selman and Pardo, 2002; Bringardner et al., 2008). These immune cells contribute to alveolar damage through the release of inflammatory factors (Bringardner et al., 2008). Immune dysregulation is a key contributor to IPF development, as evidenced by several prognostic biomarkers linked to the disease (Harrell et al., 2019). Inflammatory cytokines produced by immune cells can activate fibroblasts, promote proliferation of connective tissue cells, and facilitate angiogenesis (Jee et al., 2019). Investigating inflammatory mediators as potential biomarkers may offer valuable insights for predicting disease progression. Recent studies have reported elevated levels of inflammation-related genes and mediators in the bronchoalveolar lavage fluid (BALF) of IPF patients (Lv et al., 2019; Yin et al., 2022). Furthermore, gene expression profiles of BALF cells residing on the alveolar surface have been demonstrated to predict patient survival (Prasse et al., 2019). Consequently, some studies have developed prognostic models for IPF based on multi-gene signatures derived from BALF cells (He et al., 2022). While these findings underscore the importance of inflammation in IPF pathogenesis and progression, the specific roles and mechanistic implications of inflammation-related genes in IPF prognosis remain incompletely understood and warrant further investigation.

In this study, we analyzed mRNA microarray data from BALF samples of IPF patients obtained from the Gene Expression Omnibus (GEO) database to identify inflammation-related genes with significant differential expression. Using various machine learning methods, we constructed a risk model for IPF prognosis integrating these inflammation-related genes. Validation of this model aims to improve prognostic assessment and potentially identify novel biomarkers to guide diagnosis, treatment, and prognosis of IPF. Complementary animal experiments were performed to investigate the mechanisms associated with key hub genes implicated in IPF.

## Materials and methods

### Exploration of inflammation-related genes

We extracted 619 inflammation-related genes from Gene Ontology (GO) and GO annotations (https://www.ebi.ac.uk/QuickGO/) and compiled detailed annotation information for these genes (Supplementary Table S1). The annotation included gene function descriptions, cellular locations, and biological processes, which are crucial for understanding how these genes may influence the prognosis of IPF.

### Data collection

The publicly available GSE70866 dataset, obtained from GEO, was used for this study. This dataset includes mRNA expression data from 176 patients with IPF, along with prognostic information. In addition, we utilized the GSE175457 dataset as a validation set, comprising 188 normal samples and 234 IPF samples. Since the GSE70866 and GSE175457 dataset is publicly accessible, ethical approval from the local ethics committee was not required.

### Identifying differentially expressed inflammatory genes and functional enrichment

We analyzed the inflammation-related genes in the BALF of patients with IPF and healthy controls using the GSE70866 dataset with R’s “limma” package. A threshold was set with a false discovery rate of less than 0.05 and a log2 |fold change| greater than 1. The results were visualized using the “heatmap” and “ggplot2” packages. We further investigated the biological functions of differentially expressed inflammatory genes through GO and Kyoto Encyclopedia of Genes and Genomes (KEGG) analyses using the R packages “clusterProfiler,” “org.Hs.e.g.,.db,” “enrichplot,” and “ggplot2.” Additionally, gene set enrichment analysis (GSEA) was conducted to explore the functional enrichment of these genes.

### Weighted gene coexpression network analysis (WGCNA)

We used WGCNA to identify gene modules in IPF that exhibited strong correlations (Langfelder and Horvath, 2008). Inflammation-related genes were further analyzed by performing WGCNA using the R package. A soft thresholding power of five was selected to construct the gene network and compute the similarity and proximity in coexpression, which was then transformed into a topological overlap matrix (TOM). Based on the TOM, hierarchical clustering was applied to group the modules. Finally, we identified modules that exhibited significant associations with clinical traits.

### Developing and confirming prognostic biomarkers linked to inflammation-related genes

To minimize the risk of overfitting, we used LASSO penalized Cox regression analysis via the “glmnet” package in R to develop the prognostic model. The risk score for each patient was calculated using the regression coefficients corresponding to the normalized expression levels of prognostic inflammation-related genes. Using the median risk score as a threshold, we divided patients with IPF into two groups: high-risk and low-risk. The Kaplan–Meier survival plots and time-varying receiver operating characteristic (ROC) curves were generated using the “survival,” “survminer,” and “timeROC” R packages to assess the forecasting power of our prognostic model. Additionally, the “heatmap” software was used to visually depict gene expression patterns in each sample of the two risk groups.

### Construction of protein-protein interaction (PPI) network

To examine the interactions among these genes, we constructed a PPI network of inflammation-related genes using the STRING database. STRING, a public online resource, provides detailed information on gene and protein interactions, helping to better understand the functions and mechanisms of inflammation-related genes during the prognostic process.

### Analysis of the tumor immune response

We used the CIBERSORT method to investigate the association between prognostic genes, risk scores, and immune cell infiltration. Subsequently, the “ggplot2” package, in combination with the “limma” R package, was used to examine the relationships between hub genes, immune cells and immune checkpoints.

### Preliminary experimental validation of genes in the risk scoring model

Fifteen male Sprague–Dawley (SD) rats aged 6–8 weeks were randomly divided into the control, model, and anti-CCL2 intervention groups. Bleomycin A5 (0.2 mL of physiological saline containing 5 mg/kg) was injected into the trachea to induce the disease model, ensuring proper anesthesia to minimize stress reactions. During the modeling process, 10 μL of anti-CCL2 lentivirus solution (titer of 1 × 10^9^) was simultaneously administered through the trachea to the anti-CCL2 group. The lentivirus was used to knock down the expression of *CCL2*, with a successful infection confirmed by PCR. After 14 days of normal feeding, the rats were euthanized and weighed, and samples were collected. The entire lung was excised and weighed, and the left lungs were immediately frozen in liquid nitrogen or fixed for histological analysis.

### Quantitative polymerase chain reaction (qPCR)

Total RNA was extracted using a nuclear reaction reagent supplied by McRea-Nagel Corporation. RNA content was measured using a NanoDrop 2000 spectrophotometer (Thermo Fisher Scientific, United States). Complementary DNA (cDNA) was synthesized using the SuperScript III First-Strand Synthesis System (18080051; Invitrogen). qPCR was performed on a CFX96 qPCR system (Bio-Rad Laboratories) using a 2× SYBR Green master mix from Yeasen Biotech. The data were used to quantify the comparative mRNA expression of target genes, with GAPDH serving as the internal control. The primer sequences used were:

• Forward primer: CAG​CCA​GAT​GCA​ATC​AAT​GCC
• Reverse primer: TGG​AAT​CCT​GAA​CCC​ACT​TCT

### Protein immunoblotting analysis

To analyze protein expression in the lung tissue of IPF model rats, we employed standardized biochemical methods. Tissues were stored in liquid nitrogen, and proteins were extracted using a radioimmunoprecipitation assay buffer and quantified with a BCA Protein Assay Kit. Proteins were separated by sodium dodecyl sulfate-polyacrylamide gel electrophoresis, transferred to polyvinylidene fluoride membranes, and incubated with MCP-1-specific antibodies. Protein bands were visualized and analyzed using ImageJ software to quantify MCP-1 expression levels.

### Immunohistochemistry (IHC)

Dewaxed tissue sections were rinsed with distilled after treatment with an eco-friendly solution and ethanol. Antigens retrieval was performed by microwaving the slides in a retrieval solution, followed by natural cooling. The slides were then rinsed three times with phosphate-buffered saline (PBS), treated with 3% hydrogen peroxide (H_2_O_2_) in the dark, and washed again in PBS. Subsequently, the sections were blocked with 3% bovine serum albumin (BSA), incubated overnight at 4°C with primary antibodies, and washed in PBS. HRP-labeled secondary antibodies were added, followed by incubation. Staining was performed using 3,3’-diaminobenzidine, stopped with tap water, counterstained with hematoxylin, dehydrated, mounted, and observed under a microscope.

### Enzyme-linked immunosorbent assay (ELISA)

A total of 100 mg of tissue was weighed and homogenized with 900 μL of buffer. Standard, sample, and blank wells were set up. Subsequently, 100 μL of standards and samples were added to the respective wells. The mixture was incubated at 37°C for 1 h. After incubation, the liquid was discarded, and 100 μL of Detection Solution A was added. The wells were incubated again. The wells were washed five times, and then 100 μL of Detection Solution B was added, followed by incubation for 30 min. The wells were washed again, and 90 μL of 3,3’,5,5’-Tetramethylbenzidine substrate was added. The color change was monitored, and the optical density values were measured.

### Hematoxylin-eosin (HE) staining

For HE staining, fresh lung tissue was fixed in a fixative solution for more than 24 h to ensure adequate preservation. The tissues were trimmed, dehydrated, and embedded in paraffin. Wax blocks were sectioned into 4 μm slices. The slices were deparaffinized, stained with hematoxylin, rinsed, differentiated, and counterstained. Finally, the stained sections were mounted for observation.

### Masson trichrome staining

For Masson staining, paraffin sections were dewaxed using an environmentally friendly solution and anhydrous ethanol, then rinsed with tap water. Frozen sections were returned to room temperature and fixed in a tissue fixative. The slices were stained with Masson A solution overnight, rinsed with tap water, and stained with a mixture of Masson B and C for 1 min. Differentiation was performed, followed by immersion in Masson D for 6 min and Masson E for 1 min. The sections were then stained with Masson’s F solution, rinsed with 1% acetic acid, dehydrated, cleared, and mounted using neutral balsam.

### Data analysis

Statistical analyses were conducted using SPSS Statistics 23 (IBM, Armonk, NY, United States) and R software (version 4.3.2). For data with a normal distribution, means of continuous variables were compared using independent t-tests and are described as mean ± standard deviation. For non-normally distributed data, the Mann-Whitney U test was employed, with results presented as median [interquartile range]. Survival rate comparisons were performed using log-rank tests and Kaplan-Meier analyses. Statistical visualization was generated using GraphPad Prism 8. All tests were two-sided, with a significance level set at α = 0.05.

## Results

### Inflammation-related genes in IPF

**FIGURE 1:**
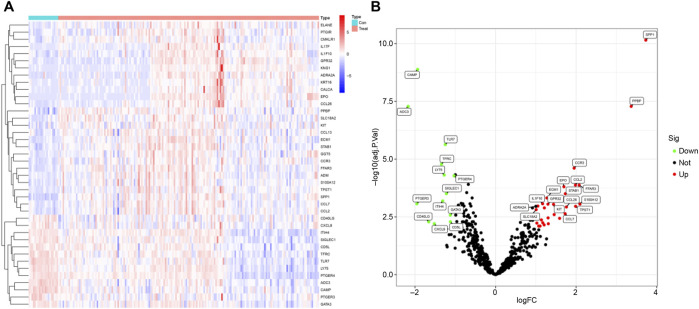
Differential Analysis. **(A)** Heatmap showing the top 20 differentially expressed genes. **(B)** Volcano plot displaying differentially expressed genes; green indicates downregulated genes, while red represents upregulated genes.

We analyzed gene expression profiles in the bronchoalveolar lavage fluid (BALF) of 176 patients with idiopathic pulmonary fibrosis (IPF) and 20 healthy controls using the GSE70866 dataset. Our investigation concentrated on inflammation-related genes, selected from the QuickGO database and comprising a total of 619 genes. The objective was to identify differentially expressed inflammation-related genes in IPF patients compared to healthy individuals, aiming to elucidate inflammatory processes underlying IPF pathogenesis. Through rigorous differential expression analysis, we identified 41 inflammation-related genes exhibiting significant expression differences between the two groups. To visually represent these results, a heatmap illustrating the top 20 differentially expressed genes was generated (Figure 1A), facilitating a comparative overview of gene expression patterns.

Furthermore, a volcano plot summarizing the differential expression analysis was constructed (Figure 1B). This plot revealed that 13 inflammation-related genes were downregulated in IPF patients, potentially reflecting suppression of specific inflammatory pathways. In contrast, 28 inflammation-related genes were upregulated, suggesting an intensified inflammatory response characteristic of IPF. These findings are important as they pinpoint specific inflammation-related genes that may contribute critically to IPF pathogenesis and progression. Identification of these genes lays the groundwork for the development of novel therapeutic strategies targeting inflammatory mechanisms, which may ultimately improve disease outcomes by mitigating inflammation-driven fibrosis.

### Functional enrichment analysis

**FIGURE 2:**
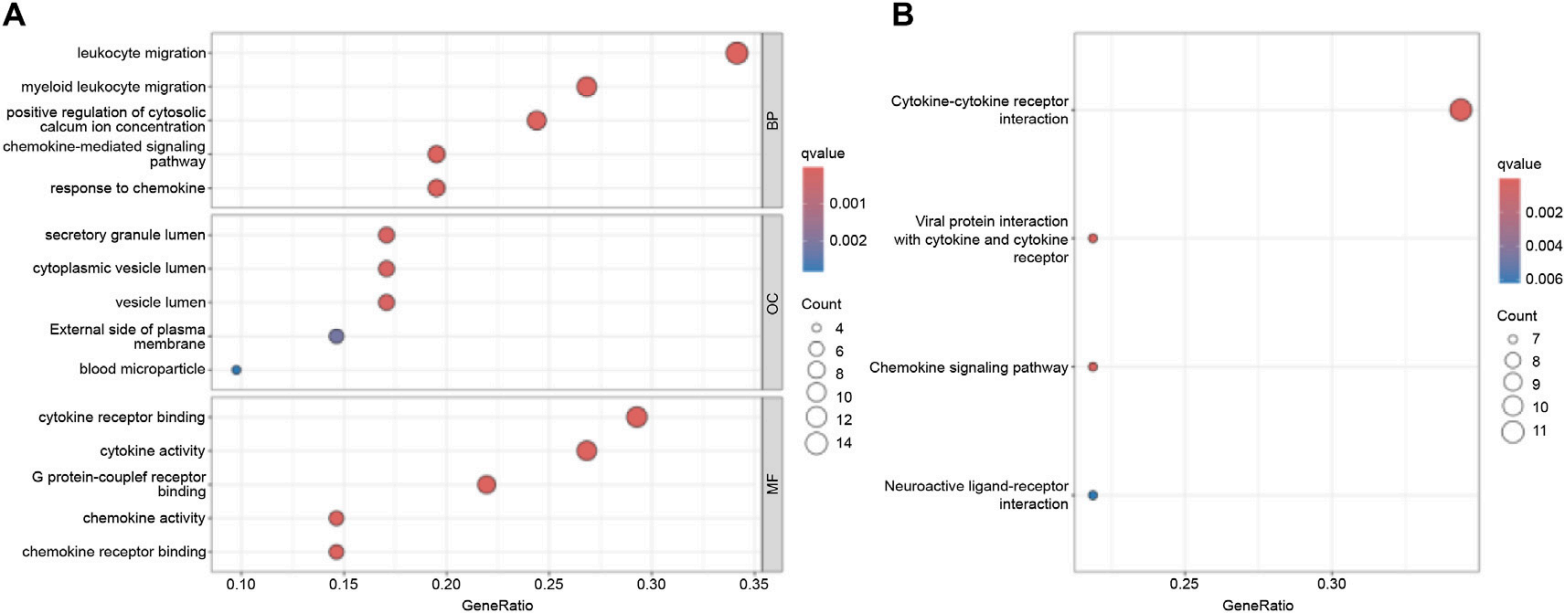
Functional Enrichment Analysis. **(A)** GO functional enrichment analysis. **(B)** KEGG pathway enrichment analysis.

GO and KEGG pathway enrichment analyses were performed to investigate the functional roles of the differentially expressed inflammation-related genes. GO enrichment analysis revealed significant overrepresentation of Biological Process (BP) terms associated with leukocyte migration. In the Cellular Component (CC) category, enriched terms included secretory granules, cytoplasmic vesicles, and vesicle lumens. Molecular Function (MF) terms showed enrichment in cytokine receptor binding, cytokine activity, and G protein-coupled receptor binding (Figure 2A). KEGG pathway analysis further highlighted significant enrichment in the cytokine–cytokine receptor interaction pathway (Figure 2B). Together, these results indicate that the differentially expressed genes participate in key biological functions and signaling pathways, providing insights into the potential mechanisms driving their involvement in IPF pathogenesis.

### Construction of predictive model

**FIGURE 3:**
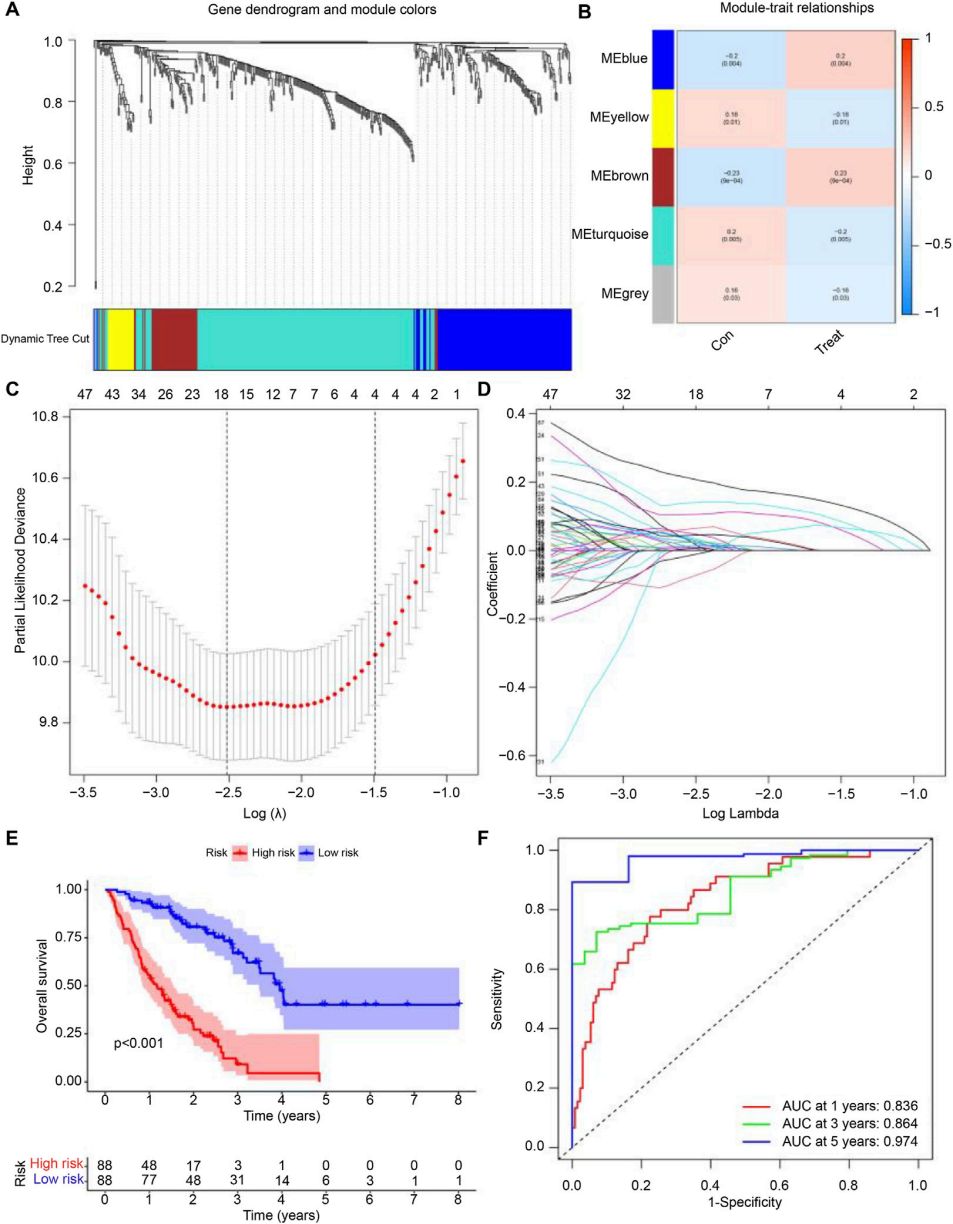
Development of the Predictive Model. **(A,B)** Correlation of feature genes from different module with IPF. **(C,D)** Gene set selected for prognostic model construction through LASSO regression analysis. **(E)** Survival curves for samples grouped by risk levels, ranging from low to high. **(F)** Time-dependent ROC curve showing the performance of the prognostic model at 1, 3, and 5 years for IPF. The false positive rate is shown on the x-axis, and the true positive rate is shown on the y-axis. The AUC value indicates the area under the curve.

We conducted WGCNA and identified the brown module as exhibiting the highest correlation with IPF (Figures 3A,B). From the genes in the brown module, we identified several hub genes associated with IPF, including *SPP1*, *CCR3*, *FFAR3*, *CCL2*, and *STAB1*. Using LASSO regression analysis, we further refined the selection and identified 18 genes suitable for constructing the prognostic model, such as *CXCR6*, *BDKRB1*, *TPST1*, *PPBP*, *CLU*, *CCL2*, *BMP6*, *LIPA*, *CXCL5*, *CAMP*, *ELF3*, *IL1RL1*, *AOC3*, *PLD4*, *S1PR3*, *CMKLR1*, *PROK2*, *STAB1* (Figures 3C,D). Based on the calculated risk scores, the IPF samples were divided into high-risk and low-risk groups. The prognostic model’s predictive ability was then evaluated through survival rate and ROC curve analyses. Kaplan-Meier analysis showed that the survival rate of the high-risk group was significantly lower than that of the low-risk group (Figure 3E). Additionally, time-dependent ROC analysis revealed that the areas under the curve (AUC) for survival rates at 1, 3, and 5 years were 0.836, 0.864, and 0.974, respectively (Figure 3F).

### Hub gene selection

**FIGURE 4:**
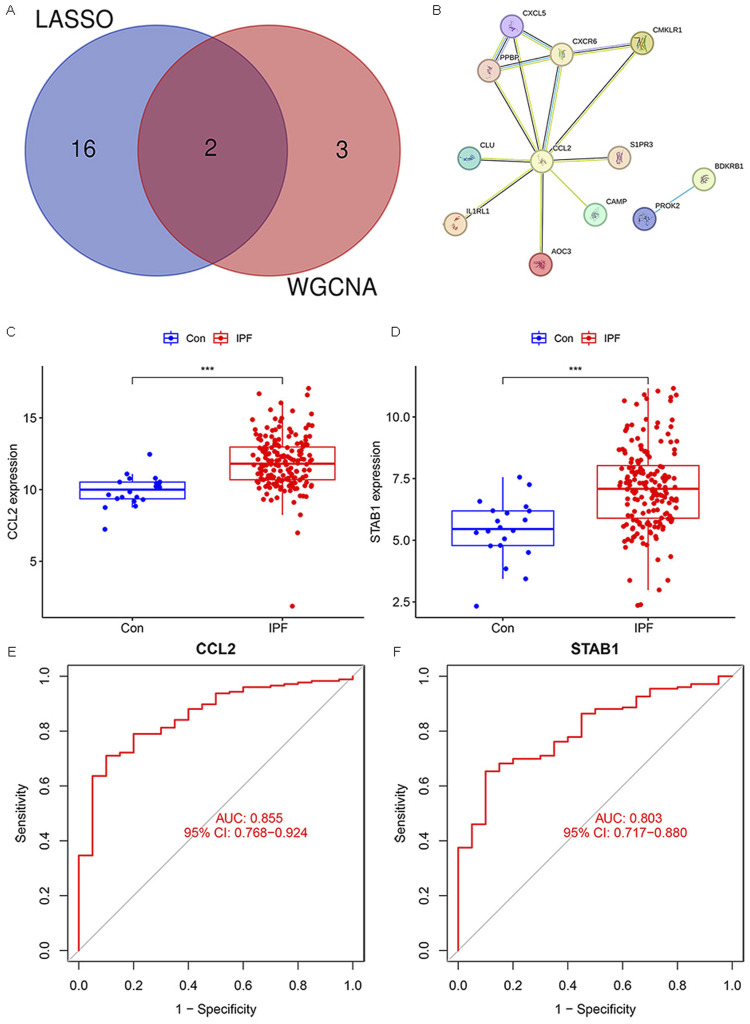
Hub Gene Selection. **(A)** Venn diagram showing overlapping genes between WGCNA and LASSO. **(B)** PPI network. **(C,D)** Box plots illustrating the expression levels of *CCL2* and *STAB1* in IPF and normal samples. **(E,F)** ROC analysis of *CCL2* and *STAB1* in IPF (***P < 0.001).

Through the intersection of WGCNA and LASSO, we identified two genes, *CCL2* and *STAB1* (Figure 4A). Based on this prognostic inflammation-related gene set, we used the STRING online platform to generate a PPI network (Figure 4B). The results revealed that the *CCL2* gene had significantly more interactions than the other genes, suggesting its potentially more crucial role. Differential analysis and ROC curve result for *CCL2* and *STAB1* showed elevated expression levels in IPF, which correlated with patient prognosis (Figures 4C–F). Finally, we used the GSE175457 dataset as a validation set to generate boxplots of *CCL2* and *STAB1* expression levels (Supplementary Figure S1). The results showed that *CCL2* expression was significantly higher in IPF samples compared to normal samples (p = 0.0038), whereas *STAB1* expression showed no significant difference (p = 0.44). Based on these findings, we focused our investigation on *CCL2*.

### Immune function and cell infiltration in high-risk and low-risk groups

**FIGURE 5:**
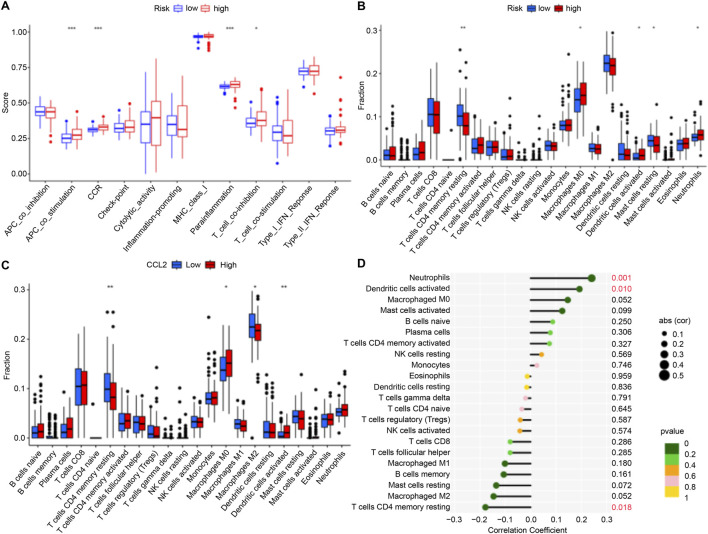
Immune Cell Infiltration. **(A,B)** Box plots illustrating variations in immune function and infiltration levels across groups with low to high risk. **(C)** Box plot illustrating differences in immune cell infiltration levels between the high-*CCL2* and low-*CCL2* expression groups. **(D)** Correlation analysis showing the relationship between *CCL2* expression levels and immune cell infiltration in patients with IPF (*P < 0.05; **P < 0.01; ***P < 0.001).

We investigated immune function and immune cell infiltration in IPF patients by comparing the high- and low-risk groups. Immune function was significantly enhanced in the high-risk group, particularly in antigen-presenting cell co-stimulation, CCR, parainflammation, and T-cell co-inhibition (Figure 5A). Immune cell infiltration analysis showed elevated levels of macrophages M0, activated dendritic cells, and neutrophils in the high-risk group, while those of T cells (CD4 memory resting) and resting mast cells were reduced (Figure 5B). These findings suggest that high-risk patients exhibit greater immune activity and immune cell infiltration.

### GSEA

**FIGURE 6:**
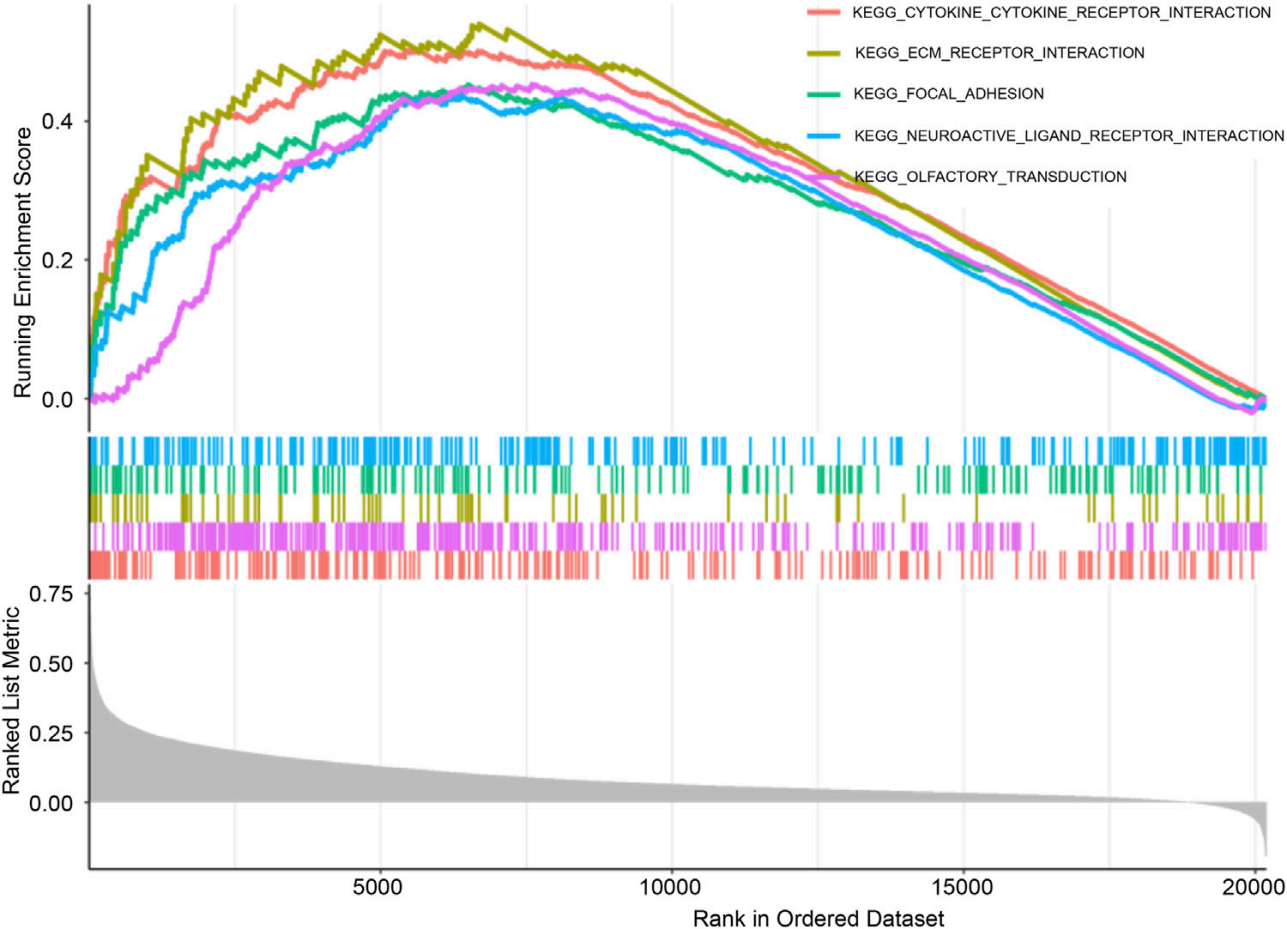
Gene set enrichment analysis.

KEGG pathway enrichment analysis was conducted using GSEA to compare high-CCL2 and low-CCL2 expression groups. The “CYTOKINE_CYTOKINE_RECEPTOR_INTERACTION” pathway demonstrated the highest enrichment in patients with high-CCL2 IPF (Figure 6). Notably, pathways strongly associated with *CCL2* expression overlapped with those enriched for differentially expressed inflammatory genes, suggesting a critical role for *CCL2* and inflammatory pathways in IPF pathogenesis.

### CCL2 and immune checkpoints

**FIGURE 7:**
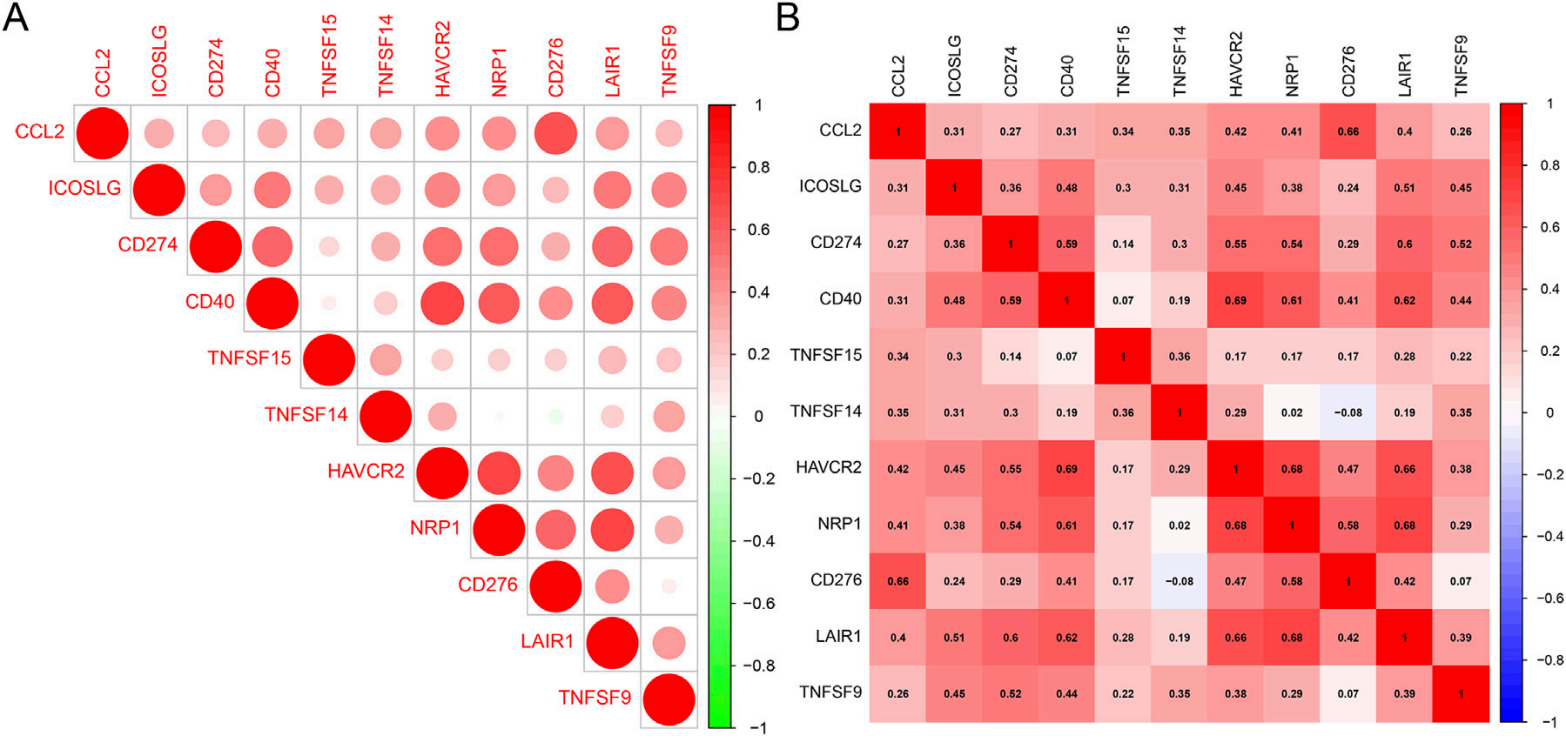
**(A, B)** Heatmap of Correlation between CCL2 and Immune Checkpoints. The red color indicates a positive correlation.

Although immune checkpoint blockade therapies have shown remarkable success, a substantial proportion of patients exhibit marked resistance (Mei et al., 2021; Nie et al., 2022). To explore the association between *CCL2* expression and immunotherapy in patients with IPF, we analyzed its relationship with various immune checkpoints. Our findings revealed that *CCL2* was significantly and positively correlated with immune checkpoints, including *CD276* and *CD40* (Figures 7A,B). These results suggest that patients in the high-*CCL2* group may benefit more from immune therapy, particularly with immune checkpoint inhibitors.

### CCL2 expression and correlation with immune cells in IPF

To validate the role of CCL2, immune cell infiltration was analyzed in IPF patients stratified by CCL2 expression levels. High CCL2 expression was associated with increased infiltration of M0 macrophages and activated dendritic cells, whereas low CCL2 expression corresponded to elevated levels of resting memory CD4^+^ T cells and M2 macrophages (Figure 5C). Correlation analysis further demonstrated a positive association between CCL2 and neutrophils as well as activated dendritic cells, alongside a negative correlation with resting memory CD4^+^ T cells (Figure 5D).

**FIGURE 8:**
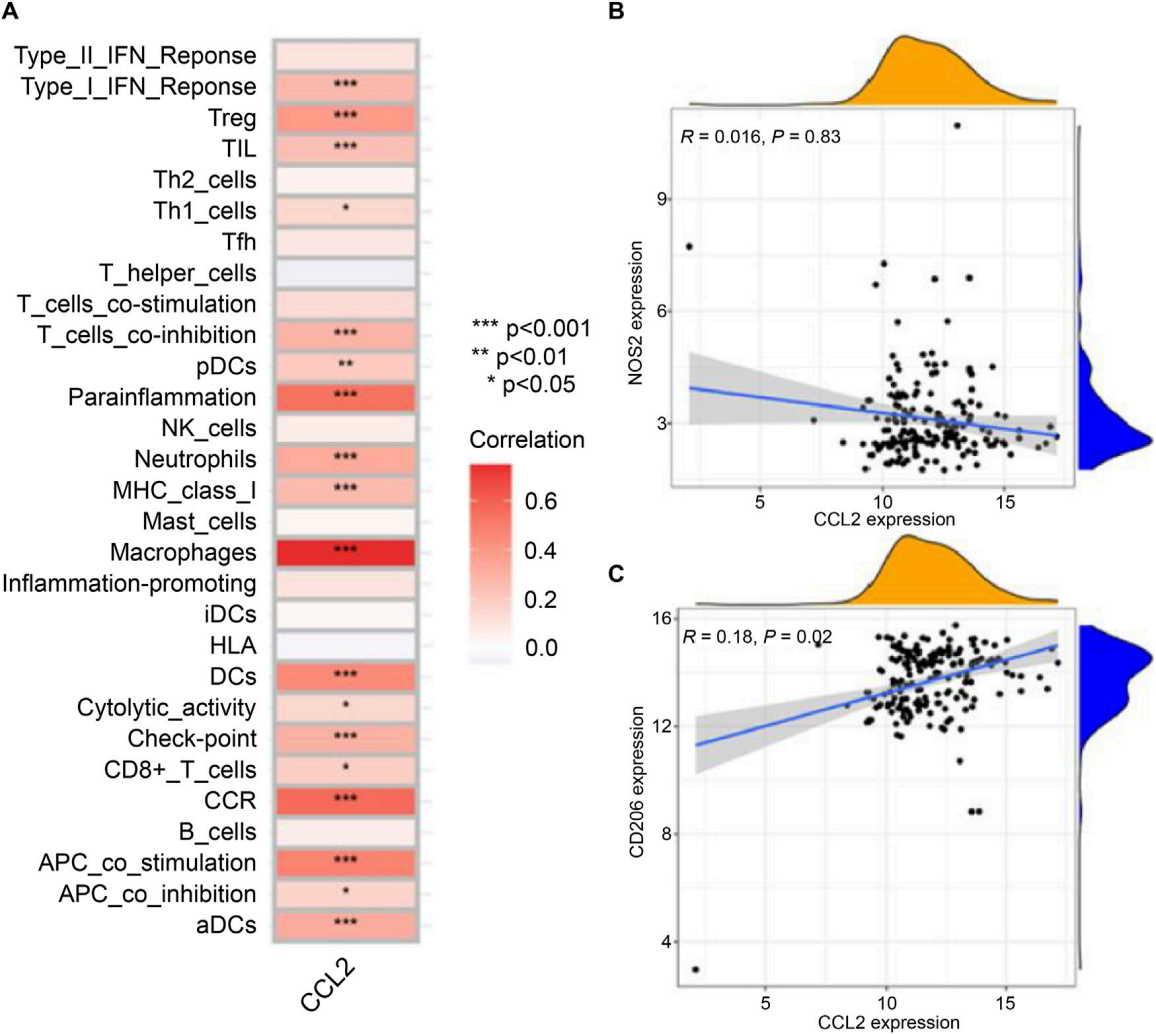
Relationship between *CCL2* and Immune Cell Infiltration. **(A)** Correlation heatmap showing the differences in infiltration levels between *CCL2* and various immune cells. **(B)** Scatter plot displaying the relationship between *CCL2* and the expression level of the macrophage M1 marker NOS2. **(C)** Scatter plot showing the association between *CCL2* and the expression level of the macrophage M2 marker CD206 (^*^P < 0.05; ^**^P < 0.01; ^***^P < 0.001).

Subsequent investigation focused on the relationship between CCL2 and macrophage polarization markers in IPF patients. CCL2 exhibited a significant positive correlation with the M2 macrophage marker CD206 (R = 0.18, p = 0.02), whereas no significant correlation was observed with the M1 macrophage marker NOS2 (R = 0.016, p = 0.83; Figures 8B,C). These findings highlight the pivotal role of CCL2 in modulating macrophage phenotypes, with its strong association with M2 macrophages implicating it in immune regulation and disease progression in IPF.

### Expression of CCL2 in rat models

**FIGURE 9:**
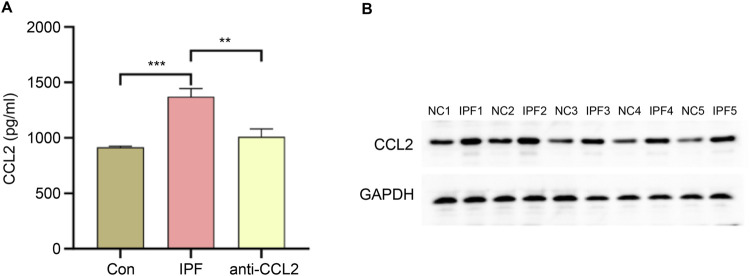
Validation of *CCL2* Expression. **(A)** qPCR comparison of *CCL2* gene expression levels in lung tissues from the control (Con) group, IPF group, and anti-*CCL2* group. **(B)** Western blot analysis comparing CCL2 protein expression levels in lung tissues from IPF and control rats (^**^P < 0.001; ^***^P < 0.0001).

To validate CCL2 expression, quantitative PCR (qPCR) and Western blot analyses were performed on lung tissue from IPF-induced rats, while qPCR was also conducted on infected rats to assess the efficiency of lentiviral transfection. As shown in Figure 9, compared to normal rat lung tissue, CCL2 expression at both the mRNA and protein levels was significantly elevated in IPF lung tissue. These results are consistent with our database analysis, supporting the notion that increased CCL2 expression may contribute to IPF pathogenesis. Furthermore, following chronic lentiviral transfection, CCL2 expression in the anti-CCL2 treatment group was significantly reduced relative to the IPF group, indicating that lentiviral transfection effectively suppresses CCL2 expression.

### HE staining and Masson’s trichrome staining

**FIGURE 10:**
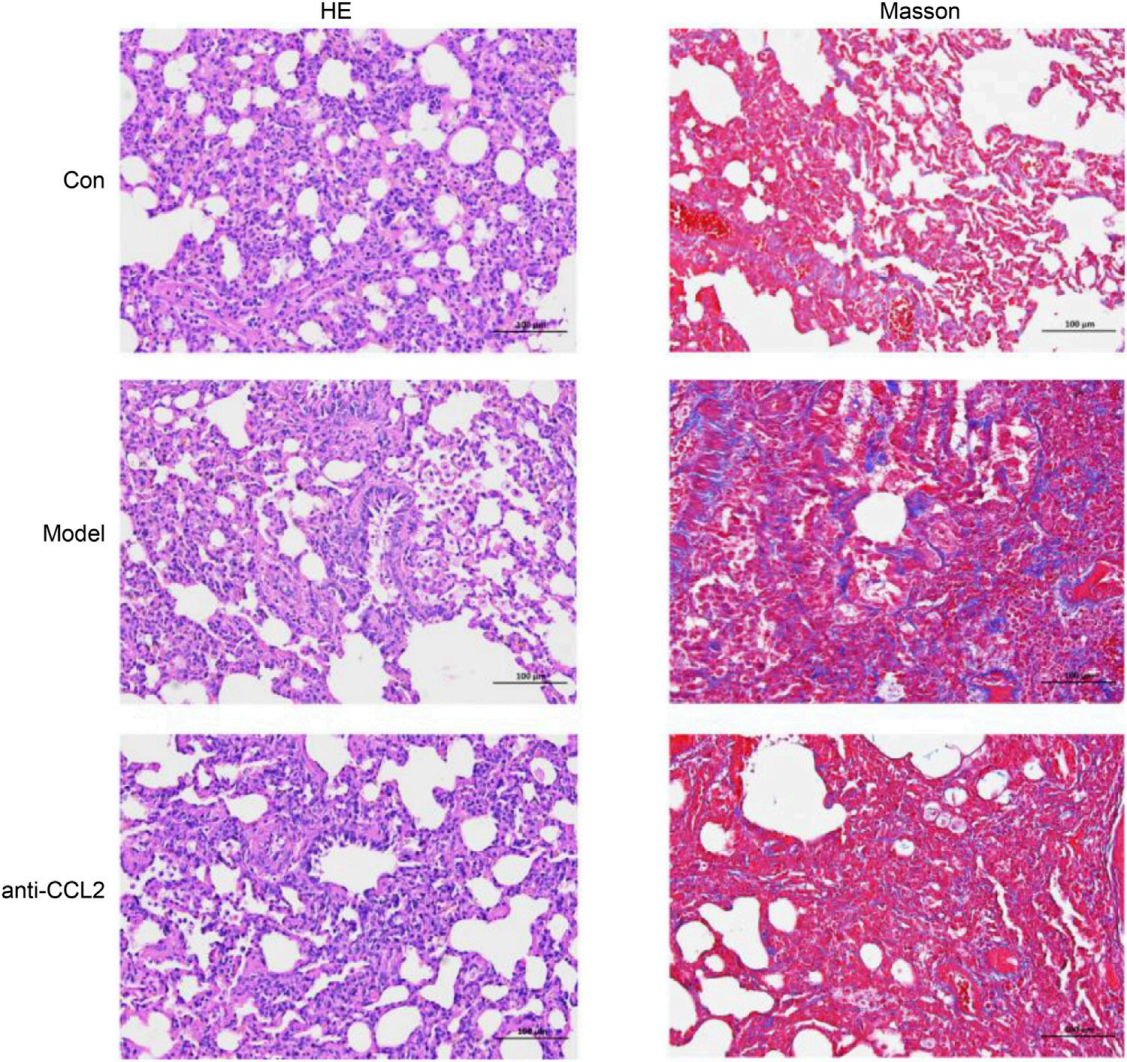
He and Masson’s staining for different groups.

To further validate the model’s effectiveness, hematoxylin and eosin (HE) and Masson’s trichrome staining were performed. HE staining provided a detailed visualization of tissue architecture, allowing assessment of inflammatory cell infiltration and structural alterations in lung tissue. In the model group, HE staining (Figure 10) revealed prominent infiltration of inflammatory cells, primarily lymphocytes, monocytes, and macrophages, indicating a pronounced chronic inflammatory response. Additionally, notable pathological features such as thickened alveolar walls and airway remodeling were observed, consistent with the hallmark characteristics of idiopathic pulmonary fibrosis (IPF). Masson’s trichrome staining was employed to evaluate collagen deposition. In the model group, a marked increase in blue-stained collagen fibers was evident, demonstrating a positive correlation between collagen accumulation and fibrosis severity. These observations align with previously reported collagen deposition patterns in IPF, underscoring the critical role of aberrant collagen accumulation in disease pathology (Figure 10). Importantly, collagen deposition was significantly reduced in the anti-CCL2 treatment group compared to the model group, suggesting that lentiviral-mediated knockdown of CCL2 partially attenuates fibrosis progression.

### ELISA and immunohistochemical analysis

**FIGURE 11:**
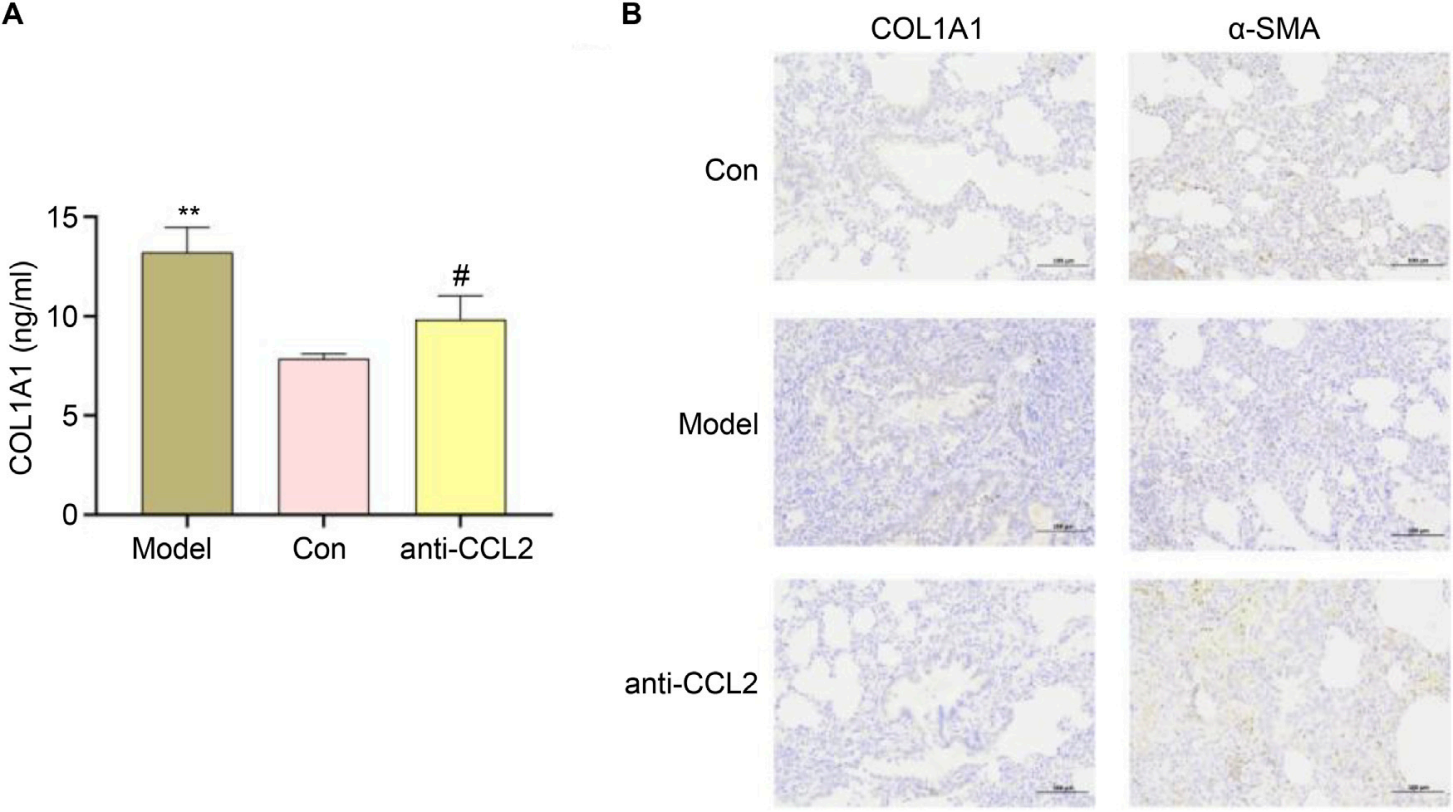
**(A)** ELISA results for different groups (^#^P < 0.01; ^**^P < 0.01). **(B)** Expression Levels of α-SMA and COL1A1.

We subsequently evaluated the successful construction of the IPF model using ELISA. In the IPF rat model, detecting COL1A1 (collagen I) holds significant biological importance. COL1A1, the most abundant collagen protein, constitutes the matrix of lung tissue and plays an important role in tissue repair and remodeling. In IPF, lung tissue undergoes abnormal fibrosis, leading to excessive deposition of COL1A1. This deposition serves as a key marker of fibrosis, reflecting the structural changes and severity of fibrosis in lung tissue. Our results demonstrated a marked increase in COL1A1 expression in the model group, indicating extensive fibrosis. Conversely, the anti-CCL2 group exhibited a significant decrease in COL1A1 expression levels (Figure 11A), suggesting that *CCL2* knockdown mitigated fibrosis. In immunohistochemical analysis, we assessed the expression levels of α-SMA and COL1A1. α-SMA, a classical marker of myofibroblasts, is typically upregulated during fibrosis. The results revealed a pronounced increase in α-SMA and COL1A1 in the model group, indicating enhanced myofibroblast transformation and collagen deposition during IPF progression (Figure 11B). Treatment with anti-CCL2 significantly reduced the expression of α-SMA and COL1A1, highlighting that CCL2 likely promotes the expression of these proteins. These findings underscore the pivotal role of CCL2 in driving the fibrosis process.

## Discussion

Idiopathic pulmonary fibrosis (IPF) is a chronic disease characterized by structural damage, fibrosis, and progressive loss of pulmonary function; however, its etiology and pathogenesis remain incompletely understood (Mei et al., 2021). Inflammation plays a critical role in the development and progression of IPF. Lung tissues from patients with IPF commonly exhibit inflammatory responses involving various pro-inflammatory factors and immune cells. These mediators contribute to alveolar tissue injury by producing inflammatory molecules that accelerate fibrosis (Nie et al., 2022; Nie et al., 2023).

This study investigated the potential roles of inflammation-related genes in IPF prognosis by analyzing gene expression profiles in bronchoalveolar lavage fluid (BALF). Our findings revealed differentially expressed inflammation-related genes in BALF from patients with IPF, several of which were associated with disease prognosis. These genes participate intricately in IPF pathology by influencing inflammation, fibrosis, and the injury and remodeling of lung structures through multiple critical pathways. Moreover, their differential expression provides insights into the role of inflammation at various stages of IPF progression, offering novel perspectives for predicting disease outcomes.

Our analysis identified CCL2 as a key gene within the inflammatory gene network in IPF (Figure 4). Previous studies have demonstrated that the development of fibrotic lesions depends on chemokines released from injured lung tissue, particularly CCL2 and CCL12 (Moore and Hogaboam, 2008). As a chemokine, the upregulation of CCL2 and CCL7 promotes the infiltration and activation of inflammatory cells during IPF progression, thereby sustaining lung inflammation (Deng et al., 2013; Krafft et al., 2013; Shinoda et al., 2009). Shinoda et al. reported that BALF levels of CCL2 were significantly elevated in IPF patients who did not survive beyond 5 years post-diagnosis compared to those with better prognoses, suggesting that elevated CCL2 predicts poorer outcomes (Shinoda et al., 2009). Similarly, our study found increased CCL2 expression in IPF patients, which may contribute to enhanced immune cell accumulation in the lungs, further promoting inflammatory infiltration and potentially worsening prognosis.

Regarding immune cell infiltration, the high-CCL2 expression group exhibited increased levels of M0 macrophages and activated dendritic cells compared to the low-CCL2 expression group. Conversely, the low-CCL2 group showed greater infiltration of resting memory CD4^+^ T cells and M2 macrophages (Figure 5C). Correlation analysis revealed a negative association between CCL2 expression and resting memory CD4^+^ T cells, and positive correlations with neutrophils and activated dendritic cells (Figure 5D). Immune dysregulation and inflammation are fundamental to the pathogenesis of chronic lung diseases, including IPF (Jee et al., 2019), and immune cell infiltration correlates with disease severity (Wang et al., 2020). CIBERSORT analysis confirmed elevated M0 macrophages and activated dendritic cells in the high-CCL2 group, indicative of heightened inflammatory activation (Li et al., 2018).

The transforming growth factor-beta (TGF-β) pathway is a central regulator of fibrosis, promoting fibroblast proliferation, myofibroblast differentiation, and extracellular matrix production, thereby playing a pivotal role in IPF. Studies have shown that CCL2 expression is upregulated in IPF lung tissue and correlates with macrophage infiltration, which may enhance TGF-β-mediated fibroblast activation (O’Donnell et al., 2019). In other fibrotic conditions such as liver fibrosis, CCL2 contributes to hepatic stellate cell activation, partially through TGF-β signaling (Car et al., 1994). These findings suggest that in IPF, CCL2 may indirectly exacerbate fibrosis by recruiting macrophages that promote TGF-β production, establishing a positive feedback loop. Additionally, the nuclear factor kappa B (NF-κB) pathway, a key regulator of inflammation, is implicated in IPF; elevated CCL2 levels in bronchoalveolar lavage fluid and lung tissue are associated with increased NF-κB activity in macrophages, suggesting that NF-κB may drive CCL2 expression in IPF (Seki et al., 2009; Deshmane et al., 2009). Collectively, these data highlight the importance of the relationship between CCL2 expression and immune cell infiltration in the inflammatory responses underlying IPF.

The identification of immune checkpoints such as programmed death-1 (PD-1) and cytotoxic T lymphocyte antigen-4 (CTLA-4) has revolutionized cancer immunotherapy (Antoniades et al., 1992). Recent immunohistochemical studies have demonstrated significantly elevated PD-1 expression in lung tissues from healthy donors, IPF patients, and lung cancer patients, with notably higher expression in IPF and lung cancer samples compared to healthy controls (Tesch et al., 2024). Moreover, preclinical rat models have shown that immune checkpoint inhibitors can reduce lung fibrosis severity (Ono et al., 2023). In this study, we observed a positive correlation between CCL2 and several immune checkpoints, suggesting that CCL2 may serve as a promising target for future immunotherapeutic strategies. Although we validated CCL2 expression in rat IPF models, the complex interactions between immune cells and immune checkpoints require further experimental exploration.

In conclusion, CCL2 appears to enhance the expression of fibrosis-related proteins COL1A1 and α-smooth muscle actin (α-SMA), implicating these proteins in mediating the pro-fibrotic effects of CCL2. COL1A1, a major extracellular matrix component, plays a critical role in various physiological and pathological processes. The increased expression of α-SMA, a key cytoskeletal protein, reflects the differentiation of fibroblasts into myofibroblasts—a hallmark of pulmonary fibrosis. Previous studies have reported a positive correlation between CCL2 and COL1A1, suggesting that COL1A1 modulates local inflammation by stimulating CCL2 synthesis. Furthermore, in liver fibrosis models, CCL2 and other pro-inflammatory markers are elevated alongside COL1A1 overexpression, indicating a synergistic role in fibrosis (Milbank et al., 2023). CCL2 recruits monocytes to sites of injury, initiating local inflammatory responses that may closely interact with α-SMA expression (Qin et al., 2020). Additionally, IL-19 has been shown to modulate α-SMA expression via CCL2 and TGF-β signaling pathways in models of diabetes-induced kidney damage, contributing to fibrosis development (Ni et al., 2018). Investigating the interplay among these molecules will deepen our understanding of disease mechanisms and may reveal novel therapeutic targets for IPF. It is worth noting that the gene expression differences between bronchoalveolar lavage fluid and lung tissue samples may reflect inherent cellular heterogeneity. While this introduces variability, it also captures distinct aspects of IPF pathophysiology, providing complementary insights. Future studies using matched samples from the same patients could help clarify these differences.

In summary, inflammation-related genes interact through multiple critical pathways influencing the pathological processes and prognosis of IPF. Based on these genes, we developed a robust prognostic model and identified CCL2 as a representative gene involved in inflammatory responses. CCL2 is closely linked to immune cell infiltration and multiple immune checkpoints in IPF, making it a promising biomarker. Moreover, CCL2 promotes the expression of COL1A1 and α-SMA, suggesting its pro-fibrotic effects are mediated, at least in part, through these proteins.

## Data Availability

Publicly available datasets were analyzed in this study. These data are accessible through the Gene Expression Omnibus (GEO) database (https://www.ncbi.nlm.nih.gov/geo/) under the accession number GSE70866.

## Abbreviations

ROC, Receiver Operating Characteristic; AUC, Area Under Curve; GO, Gene ontology; KEGG, Kyoto Encyclopedia of Genes and Genome; PPI, Protein-protein interaction; GEO, Gene Expression Omnibus; IPF, Idiopathic Pulmonary Fibrosis; WGCNA, Weighted Gene Co-expression Network Analysis; LASSO, least absolute shrinkage and selection operator.
