# Supplementary figures and images for: Role of inflammation-related genes as prognostic biomarkers and mechanistic implications in idiopathic pulmonary fibrosis
Source: Front Genet. 2025 Jun 18;16:1602588. doi: 10.3389/fgene.2025.1602588 (PMC12213808; doi:10.3389/fgene.2025.1602588)

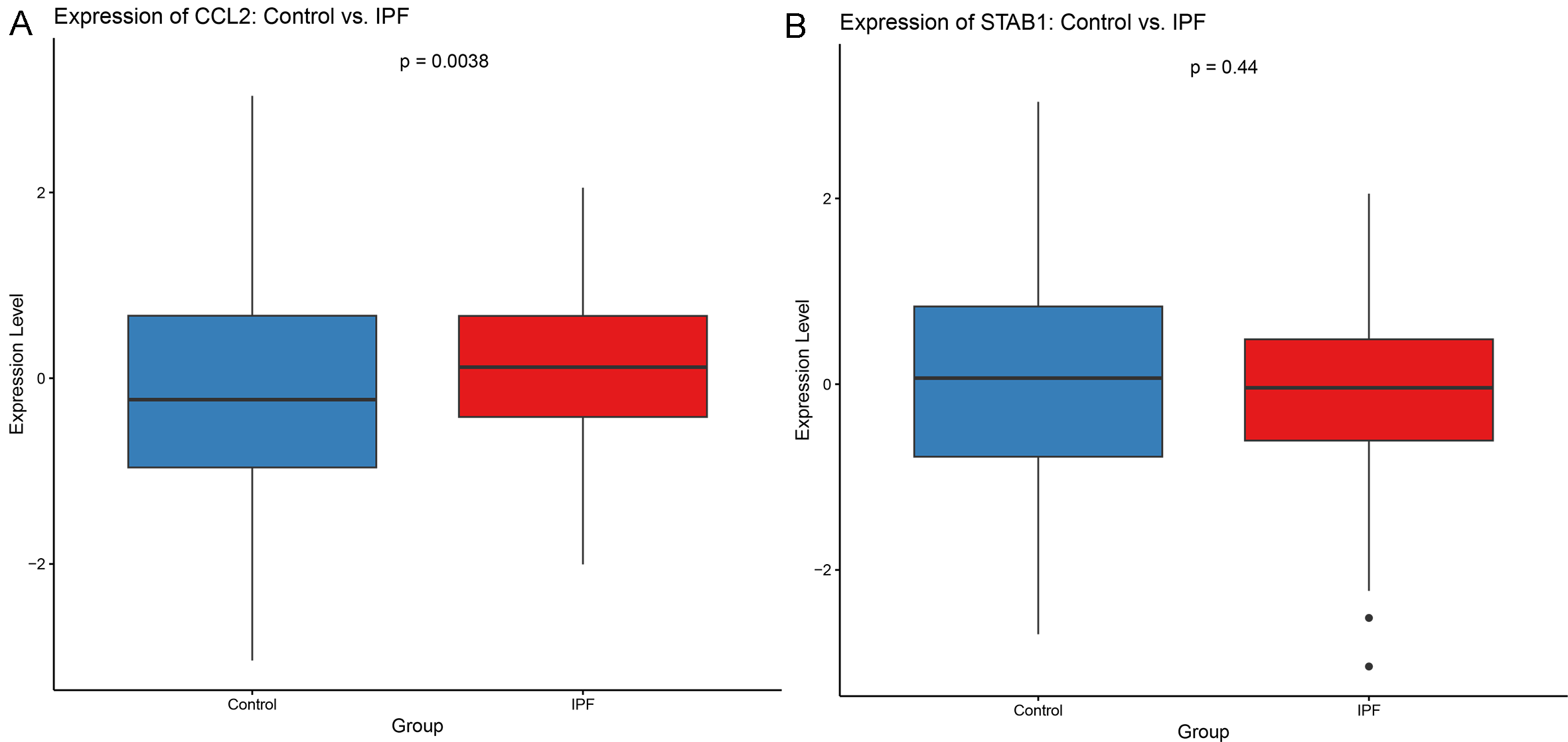

Supplement: Supplementary file 1 [file Image1.tif]
